# Solar Energy

**Published:** 1882-04

**Authors:** 


### SOLAR ENERGY.

Dr. C. W. Siemens, in a paper on the conservation of solar energy, contends that our solar system would no longer impress us with the idea of prodigious waste through dissipation of energy into space, but rather with that of well-ordered, self-sustaining action, capable of perpetuating solar radiation to the remotest future, if the following conditions actually prevail : 1. That aqueous vapor and carbon compounds are present in stellar or inter-planetary space. 2. That these gaseous compounds are capable of being dissociated by the sun's energy while they are in a state of extreme attenuation. 3. That these dissociated vapors are capable of being compressed into the solar photosphere by a process of interchange with an equal amount of reassociated vapors, this interchange being effected by the centrifugal action of the sun itself.

